# Simultaneous Disruption of Two DNA Polymerases, Polη and Polζ, in Avian DT40 Cells Unmasks the Role of Polη in Cellular Response to Various DNA Lesions

**DOI:** 10.1371/journal.pgen.1001151

**Published:** 2010-10-07

**Authors:** Kouji Hirota, Eiichiro Sonoda, Takuo Kawamoto, Akira Motegi, Chikahide Masutani, Fumio Hanaoka, Dávid Szüts, Shigenori Iwai, Julian E. Sale, Alan Lehmann, Shunichi Takeda

**Affiliations:** 1 CREST Research Project, Japan Science and Technology, Radiation Genetics, Graduate School of Medicine, Kyoto University, Kyoto, Japan; 2 Solution-Oriented Research for Science and Technology (SORST), Japan Science and Technology Agency, Graduate School of Frontier Biosciences, Osaka University, Osaka, Japan; 3 St. George's, University of London, London, United Kingdom; 4 Division of Chemistry, Graduate School of Engineering Science, Osaka University, Osaka, Japan; 5 Medical Research Council Laboratory of Molecular Biology, Cambridge, United Kingdom; 6 Genome Damage and Stability Centre, University of Sussex, Brighton, United Kingdom; Brandeis University, United States of America

## Abstract

Replicative DNA polymerases are frequently stalled by DNA lesions. The resulting replication blockage is released by homologous recombination (HR) and translesion DNA synthesis (TLS). TLS employs specialized TLS polymerases to bypass DNA lesions. We provide striking *in vivo* evidence of the cooperation between DNA polymerase η, which is mutated in the variant form of the cancer predisposition disorder xeroderma pigmentosum (XP-V), and DNA polymerase ζ by generating *POLη^−/−^/POLζ^−/−^* cells from the chicken DT40 cell line. *POLζ^−/−^* cells are hypersensitive to a very wide range of DNA damaging agents, whereas XP-V cells exhibit moderate sensitivity to ultraviolet light (UV) only in the presence of caffeine treatment and exhibit no significant sensitivity to any other damaging agents. It is therefore widely believed that Polη plays a very specific role in cellular tolerance to UV-induced DNA damage. The evidence we present challenges this assumption. The phenotypic analysis of *POLη^−/−^/POLζ^−/−^* cells shows that, unexpectedly, the loss of Polη significantly rescued all mutant phenotypes of *POLζ^−/−^* cells and results in the restoration of the DNA damage tolerance by a backup pathway including HR. Taken together, Polη contributes to a much wide range of TLS events than had been predicted by the phenotype of XP-V cells.

## Author Summary

DNA replication is a fragile biochemical reaction, as the replicative DNA polymerases are readily stalled by DNA lesions. The resulting replication blockage is released by translesion DNA synthesis (TLS), which employs specialized TLS polymerases to bypass DNA lesions. There are at least seven TLS polymerases known in vertebrates. However, how they cooperate *in vivo* remains one of central questions in the field. We analyzed this functional interaction by genetically disrupting two of major TLS polymerases, Polη and Polζ, in the unique genetic model organism, chicken DT40 cells. Currently, it is widely believed that Polη plays a very specific role in cellular tolerance to ultraviolet light–induced DNA damage. Polζ, on the other hand, plays a key role in cellular tolerance to a very wide range of DNA–damaging agents, as *POLζ^−/−^* cells are hypersensitivity to a number of DNA damaging agents. Our phenotypic analysis of *POLη^−/−^/POLζ^−/−^* cells shows that, unexpectedly, the loss of Polη significantly rescued all mutant phenotypes of *POLζ^−/−^* cells. The genetic interaction shown here reveals a previously unappreciated role of human Polη in cellular response to a wide variety of DNA lesions and two-step collaborative action of Polymerase η and ζ.

## Introduction

DNA replication involves a rapid but fragile enzymatic mechanism that is frequently stalled by damage in the DNA template. To complete DNA replication, DNA lesions are bypassed by specialized DNA polymerases, a process called translesion synthesis (TLS) (reviewed in [1], [2]). A number of TLS polymerases, including Polη and Polζ, that are conserved throughout eukaryotic evolution, have been identified in yeast and mammals. Polη deficiency is responsible for a variant form of xeroderma pigmentosum (XP-V) [3], [4] that is characterized by UV photosensitivity and a predisposition to skin cancer (reviewed in [5]). Deficiency in Rev3, the catalytic subunit of Polζ, results in a considerably more severe phenotype, compared with Polη. In fact, Rev3 disruption is lethal to mouse embryogenesis [6]. Chicken DT40 cells deficient in Rev3 exhibit significant chromosome instability and hypersensitivity to a wide variety of DNA-damaging agents [7]–[10]. In addition to their role in TLS, both Polη and Polζ can contribute to homologous DNA recombination (HR) in DT40 cells [9], [11], [12].

Exposure to UV induces cyclobutane pyrimidine dimers (CPDs) and 6-4 UV photoproducts in DNA. While Polη can efficiently and accurately bypass CPDs [4], [13]–[15], no single DNA polymerase has been shown to be capable of effectively bypassing 6-4 UV photoproducts *in vitro*. This suggests that the coordinate use of more than one polymerase is required to bypass such damage *in vivo*. In support of this notion, several biochemical studies have suggested that lesion bypass can be effected by two sequential nucleotide incorporation events [16], [17]. For example, to bypass the 6-4 UV photoproduct, Polη inserts a nucleotide opposite the damage as a first step, followed by extension from the inserted nucleotide as a second step. This extension process has been shown to be catalyzed by yeast Polζ and by human Polκ [1], [2], [18]. The contribution of mammalian Polζ to the extension step remains elusive, because functional Polζ has not been purified [19], [20]. Recently, replication of episomal plasmid DNA carrying various lesions was analyzed in mammalian cell lines to define the role of each DNA polymerase in TLS past individual DNA lesions [15], [21], [22]. Shachar et al. suggest the sequential usage of Polη and Polζ in TLS past the cisPt-GG lesion [21], while results of others do not support this two-step TLS model [15], [23]. Indeed, evidence for the two-step model in the replication of chromosomal DNA has so far been lacking. By contrast, both human and yeast Polη can bypass CPDs effectively *in vitro* without extension polymerases [4].

Combining genetic tractability with a number of sensitive phenotypic assays, the chicken DT40 B lymphocyte cell line provides a unique opportunity to precisely analyze the role of individual DNA polymerases in TLS as well as in HR. The immunoglobulin loci of DT40 cells undergo constitutive diversification in culture by a combination of gene conversion (which depends on HR) and point mutation (which depends on TLS [24]). This diversification is driven by activation-induced deaminase (AID) [25], [26], which catalyzes the deamination of cytosine to generate uracil in the immunoglobulin loci. The uracil is then eliminated by uracil glycosylase to form abasic sites, which are thought to be the lesions that trigger bypass, either by gene conversion or by mutagenic translesion synthesis (reviewed in [27]). To study TLS in a different context, an episomal plasmid-based system was recently developed to examine the replication of a plasmid carrying site-specific lesions, in this case 6-4 UV photoproducts, in DT40 cells [28].

To investigate the functional interaction between Polη and Polζ in the DT40 cell line, we created *POLη^−/−^/POLζ^−/−^* DT40 cells (hereafter called *polη/polζ* cells). Unexpectedly, depletion of Polη in the *polζ* cells suppressed virtually all mutant phenotypes associated with the loss of Polζ, including genome instability and hypersensitivity to DNA-damaging agents. Furthermore, the reconstitution of *POLη^−/−^/POLζ^−/−^* cells with intact human Polη, but not the polymerase-deficient mutant carrying D115A/E116A substitutions, increased their hypersensitivity to DNA-damaging agents to the level of the *POLζ^−/−^* cells, indicating that Polη-dependent DNA synthesis is toxic in the absence of Polζ. Remarkably, this alleviation of the *polζ* phenotype was associated with the restoration of effective translesion synthesis. These data provide *in vivo* support for the two-step model of lesion bypass, with Polζ playing a critical role in the extension step following nucleotide incorporation by Polη.

## Results

### Deletion of Polη reversed the hypersensitivity of the *polζ* mutant to UV, ionizing radiation, MMS, and cisplatin

**Figure 1:**
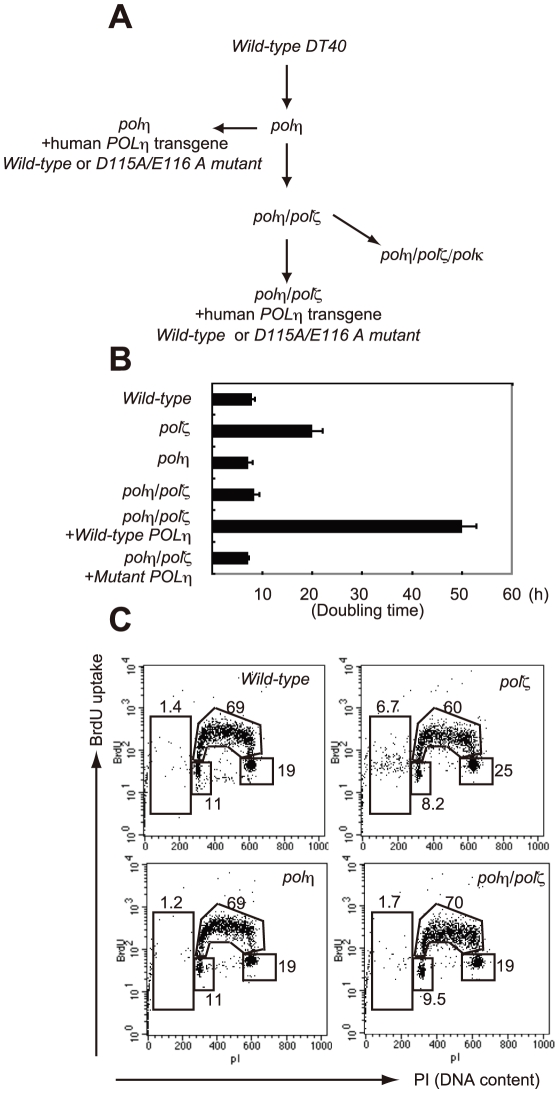
Proliferation of chicken DT40 *polζ*, *polη*, and *polη/polζ* cells. (A) Schematic representation of the generation of the mutant cells used in this study. (B) The *polη* and *polη/polζ* mutants grew more rapidly than did the *polζ* cells. Cells were cultured for 3 days. The doubling time of each mutant is shown. Note that *wild-type* cells divide every 8 hours. “+*Wild-type POLη*” represents a reconstitution of *polη/polζ* cells with *wild-type* human *polη*. “*Mutant POLη*” carries D115A/E116A mutations, which inactivate the polymerase activity of human Polη. Error bars show the SD of the mean for three independent assays. (C) Representative asynchronous flow cytometric cell-cycle profiles of *wild-type*, *polη polζ*, and *polη/polζ* cells. Cells were pulse-labeled with BrdU for 10 minutes and stained with propidium iodide (PI) to measure DNA content. The large gate on the left of each panel indicates the sub-G_1_ apoptotic cell fraction. The lower left, arch, and lower right gates indicate cells in the G_1_, S and G_2_/M phase, respectively. Numbers show the percentage of cells falling in each gate.

We generated *polη/polζ* cells by inactivating both *REV3* alleles of the *polη* DT40 cells using a previously published gene-targeting strategy (Figure 1A) [9], [12]. The growth properties of the mutant cells were examined by measuring their growth rate and cell-cycle profile. As reported previously, the *polη* cells had a normal growth rate, whereas the *polζ* cells proliferated more slowly, exhibiting an increase in the sub-G_1_ fraction, indicative of spontaneous cell death during the cell cycle (Figure 1C). The loss of Polζ caused a significant increase in the number of spontaneous arising γH2AX foci, which represent replication collapse (Figure S1). Interestingly, deletion of *POLη* in the *polζ* cells reversed their growth retardation and reduced the rate of cell death (Figure 1B and 1C). Similarly, the number of spontaneous chromosomal aberrations was significantly reduced in *polη/polζ* cells, compared with *polζ* cells (Table 1). Ectopic expression of human Polη in *polη/polζ* cells diminished their growth rate to the level of *polζ* cells. This observation does not reflect general toxicity of the overexpressed human Polη, since its ectopic expression caused pronounced growth retardation only in the *polη/polζ* double mutant but not in *wild-type* cells (Figure S2). These observations indicate that the growth defect of *polζ* cells is dependent on the presence of Polη.

**Table 1:** Frequency of spontaneous chromosomal aberrations in *wild-type*, *polζ*, *polη*, and *polη/polζ* double mutants.*

Cell	No. of cells analyzed	Chromatid		Isochromatid		Total (per cell±SE)
		Gaps	Breaks	Gaps	Breaks	
*Wild-type*	100	0	1	1	0	2 (0.02±0.01)
*polζ*	100	0	2	2	8	12 (0.12±0.03)
*polη*	100	1	0	0	0	1 (0.01±0.01)
*polη/polζ*	100	0	0	0	2	2 (0.02±0.01)
*polη/polζ*+ *wild-type Polη*	100	0	6	1	7	14 (0.14±0.04)
*polη/polζ*+ *mutant Polη*	100	0	2	2	0	4 (0.04±0.02)

*For each preparation, 100 metaphase spreads were analyzed. The number of aberrations per cell ± SE was calculated as x/N±√x/N, based on the Poisson distribution of chromosomal aberrations. The number of cells analyzed and total aberrations were defined as N and x, respectively.

**Figure 2:**
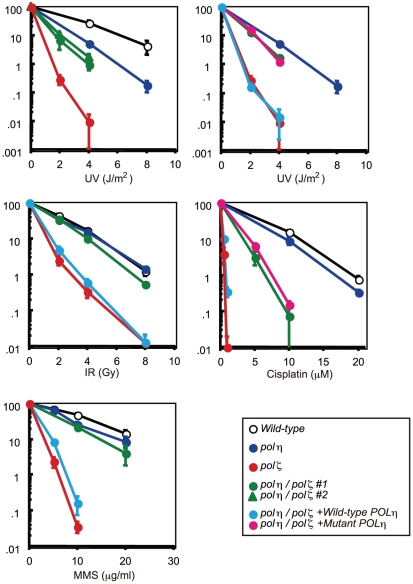
Increased tolerance of *polη/polζ* cells to various genotoxic stresses, compared with *polζ* cells. Colony survival of the indicated genotypes following the indicated genotoxic stresses. Data for the *polη/polζ* #1 clone are shown in the other figures. Error bars show the SD of the mean for three independent assays.

The sensitivity of *polη/polζ* cells to genotoxic stresses was evaluated using a colony formation assay. *polη* cells showed a mild sensitivity to UV but not to the other genotoxic stresses, in agreement with the phenotype of mammalian XP-V cells [29], [30]. In contrast, *polζ* cells showed a marked sensitivity to UV, ionizing radiation, *cis*-diaminedichloroplatinum-II (cisplatin), and methylmethane sulfonate (MMS) (Figure 2), as previously described [9]. The *polη/polζ* mutant cells were less sensitive to UV than were the *polζ* cells. Furthermore, this double mutant showed significantly increased tolerance to ionizing radiation, cisplatin, and MMS, compared with the *polζ* cells. This increased tolerance of the *polη/polζ* cells was reversed by ectopic expression of human Polη. To investigate whether this reversion depended on the polymerase activity of human Polη, we expressed human *POLη* cDNA carrying D115A/E116A mutations in the *polη/polζ* cells. These mutations in the catalytic site abolish polymerase activity (data not shown), as previously reported in yeast [31]. The expression of the mutant Polη had no effect on the sensitivity of the *polη/polζ* cells to the DNA-damaging agents (Figure 2), indicating that Polη-dependent DNA synthesis sensitizes *polζ* cells to these DNA-damaging agents.

### *polζ* cells have a more prominent defect in TLS past abasic site than *polη/polζ* cells

We wished to investigate if Polη and Polζ could collaborate in TLS past specific types of DNA damage. To this end, we performed two sets of experiments: analysis of immunoglobulin hypermutation, which in DT40 provides a readout of the bypass of abasic sites [24], and analysis of the replication of a T-T (6-4) photoproduct on an episomal plasmid [28].

**Figure 3:**
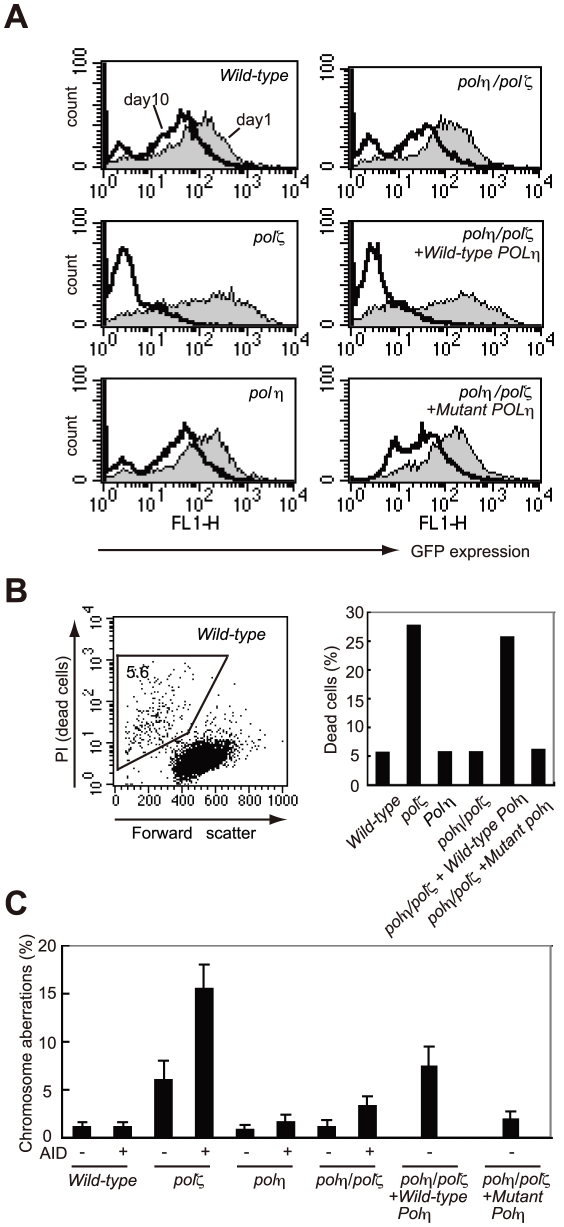
*polζ* cells, but not *polη* or *polη*/*polζ* cells, are hypersensitive to AID overexpression. (A) Reduced AID transgene expression over time in *polζ* cells. The expression of AID was estimated by measuring the intensity of the GFP signal. Filled and open distributions represent GFP intensity at days 1 and 10 post-infection, respectively. (B) Accumulation of dead cells in the *polζ* mutant population 3 days after the AID virus infection. The dot plot shows cells stained with PI. The percentage of dead cells falling into the indicated gate is shown in the histogram. (C) Chromosomal aberrations are induced by AID overexpression in *polζ* mutants. At day 3 post-infection, chromosomal aberrations in mitotic cells of the indicated genotype were analyzed. Fifty metaphase spreads were measured. The number of aberrations per cell ± SE was calculated as x/N±√x/N, where N and x are the numbers of cells analyzed and total aberrations, respectively.

To induce Ig hypermutation, we overproduced AID in DT40 cells using a retrovirus vector [32], [33]. This vector drives the monocistronic expression of AID and green fluorescent protein (GFP), allowing a comparative assessment of the level of ectopic AID expression. At 24 hours after infection with the AID retrovirus, virtually all cells from each line displayed a strong GFP signal, indicating that the deficiency of Polη and Polζ did not affect the expression of AID (Figure 3A). However, a substantial fraction of the *polζ* cells died at day 3, and with the surviving *polζ* cells at day 10 showed a decrease level of GFP signals (Figure 3A and 3B). Furthermore, *polζ* cells, but not *polη* or *polη/polζ* cells, displayed prominent chromosomal breaks at day 3 post-infection (Figure 3C). Since the break sites on the chromosome were randomly distributed, the overexpressed AID protein may be targeting a number of different loci in addition to the Ig locus, in DT40 cells. These observations suggest that TLS past abasic sites created by the combined action of AID and uracil glycosylase may be performed less effectively in *polζ* cells, compared with *polη/polζ* or *polη* cells, resulting in chromosome breakage and cell death.

To verify that Polη-dependent DNA synthesis was toxic to the AID-overproducing *polζ* cells, we reconstituted *polη/polζ* cells with either *wild-type POLη* or the catalytically inactive mutant *polη*D115A/E116A). *Wild-type POLη* expression sensitized the *polη/polζ* cells to the overexpression of AID, whereas the mutant *POLη* had no impact on cell survival (Figure 3A and 3B). We thus conclude that, in *polζ* cells, TLS past abasic sites may be less effective because neither Polη nor any other polymerase can extend DNA synthesis following the Polη-mediated insertion of nucleotides opposite the abasic site.

**Figure 4:**
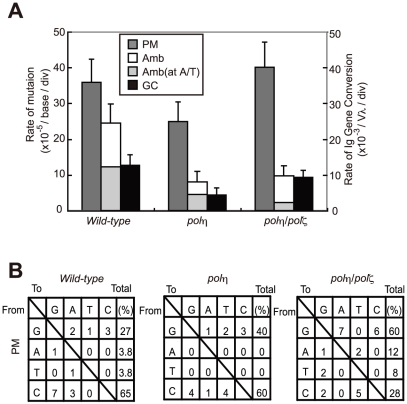
TLS–dependent point mutations in Ig V occur with comparable frequency in *wild-type* and *polη/polζ* cells. (A) Rate of non-templated single-base substitutions (PM), ambiguous single base substitutions (Amb) (which are single base changes that have a potential donor sequence in the pseudogene array), and *bona fide* gene conversion tracts (GC) in the AID-virus-infected *wild-type*, *polη*, and *polη/polζ* DT40 cells shown in Figure 3. Cells were clonally expanded for two weeks. Three clones were analyzed in each data set. Note that the Amb mutation at A/T pairs represents short-tract gene conversion [32]. (B) Nucleotide substitution preferences (as a percentage of the indicated number in the total mutations) deduced from the V_λ_ sequence analysis of the clones shown in (A). The preferences are shown for mutations categorized as non-templated base substitutions (PM), which are caused by TLS past abasic sites [32].

We have shown that AID overexpression results in increased TLS-mediated hypermutation at G/C base pairs in Ig V segments [32]. To define the role of Polη and Polζ in this TLS process, we determined the Ig V_λ_ nucleotide sequences of AID-overexpressing *wild-type*, *polη*, and *polη/polζ* cells. *polζ* cells were not analyzed because of the difficulty of ectopically expressing AID to the same extent as in the other lines. The number of non-templated point mutations (PM, Figure 4A) was somewhat lower in *polη* cells than in *wild-type* cells (This slight reduction is not significant (p = 0.15, t-test) [32]). Surprisingly, the level of Ig V mutations in *polη/polζ* cells was comparable to that of *wild-type* cells. This observation is in marked contrast with the fact that Rev1, an essential factor for the function of Polζ [20], plays a critical role in non-templated point mutations at the abasic site [34]. Moreover, Polζ played the critical role in cellular tolerance to AID-mediated abasic sites (Figure 3). These observations indicate that in the absence of both Polη and Polζ, other unidentified DNA polymerase(s) can participate in TLS past the abasic site. As the *polη/polζ* cells displayed a significant increase in the proportion of G/C to A/T transitions in the non-templated Ig V mutations (12/25; 48%), compared with *wild-type* cells (2/18; 11%)(Figure 4B), this unidentified DNA polymerase(s) may preferentially incorporate adenine opposite the abasic site in the absence of both Polη and Polζ.

### Lack of TLS past the T-T (6-4) UV photoproduct in *polζ* cells is reversed by the inactivation of Polη

**Figure 5:**
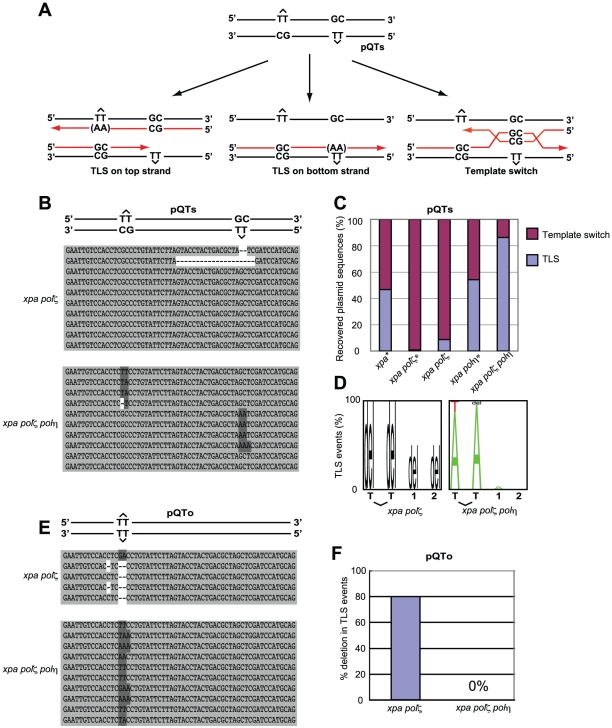
TLS past a T-T (6-4) UV photoproduct on episomal plasmid DNA. (A) Schematic of the staggered arrangement of T–T (6–4) UV photoproducts in the construct pQTs. The dinucleotide GC is placed opposite each lesion, and there are 28 bp between the lesions, with the possible outcomes of DNA replication over the area. TLS may occur on either the top or the bottom strand, with the most common base insertion shown as AA. Alternatively, the nascent strand of the sister chromatid may be used as an alternative undamaged template; one possible mechanism for such a template switching illustrated. (B) Example sequences of replicated pQTs plasmids recovered from *xpa/polζ* and *xpa/polη/polζ* clones, aligned with a schematic drawing of pQTs. TLS on the top strand (inserting mostly AA in the reverse direction, top set), TLS on the bottom strand (middle set), and error-free bypass (bottom set) are shown. Proportions are not representative. (C) The proportion of TLS versus error-free bypass in pQTs sequences recovered from *xpa/polζ* and *xpa/polη/polζ* cells, shown as a percentage of the total. Data shown with asterisks are taken from Szüts et al. [28]. A total of 23 and 29 sequences from *xpa/polζ* and *xpa/polη/polζ* cells, respectively, are shown as the sum of three independent experiments. (D) The pattern of nucleotide incorporation opposite the T-T (6-4) photoproduct in *xpa/polζ* and *xpa/polη/polζ* cells. Error-free pQTs sequences were excluded from analysis. The percentage of each nucleotide incorporated at each position is indicated by the size of the letter of the nucleotide in the column. del = deletion. The incorporation positions indicated are at the 3′ T and the 5′ T of the lesion, followed by the next two bases in the template, indicated by 1 and 2. (E) Example sequences of replicated pQTo plasmids recovered from *xpa/polζ* and *xpa/polη/polζ* clones, aligned with a schematic drawing of pQTo. (F) The frequency of replication associated with two or more nucleotide deletions in replicated copies of pQTo, which contains T–T (6–4) UV photoproducts in the opposing arrangement.

T-T (6-4) UV photoproducts represent the most formidable challenge to DNA replication, as they potently arrest replicative polymerases [35]. To analyze TLS past a T-T (6-4) photoproduct, we transfected two plasmids, pQTs and pQTo, [28] (Figure 5) carrying T-T (6-4) UV photoproducts into DT40 cells. At two days after transfection we recovered only replicated copies, and were thus able to analyze the replication of site-specific T-T (6-4) UV photoproducts *in vivo*. This photoproduct can be arranged in one of two ways. In the staggered conformation (pQTs) (Figure 5A), the lesions are separated by 28 intervening nucleotides and placed opposite a GpC mismatch. Replicated copies can thus result from TLS on the top or bottom strand. Error-free template switching should result in GpC at the site of the photoproduct, while TLS past this photoproduct may insert ApA (accurate TLS) or other nucleotides (inaccurate TLS) at this site. Note that our experiment was done in a nucleotide-excision repair-deficient (*xpa*-deficient) background, and thus excluded the recovery of replicated copies generated by error-free nucleotide-excision repair. In the second, unphysiological, replication template, the lesions are placed opposite to each other (pQTo) (Figure 5E). Using this conformation, replicated DNA copies can be recovered as a consequence of TLS, but not by template switching.

To assess the mode of bypass used when generating replicated copies of pQTs, we analyzed the nucleotide sequences of replicated copies of plasmids recovered from DT40 cells (Figure 5B) and determined the proportion of TLS relative to error-free template switching (Figure 5C). Overall replication efficiency was comparable among cells carrying the various genotypes used in the previous study and this study. Previous study found that 45% and 55% of the recovered plasmid copies resulted from TLS in *xpa* and *xpa/polη* cells, respectively [28]. In comparison, the efficiency of TLS in *xpa/polζ* cells was significantly reduced, with less than 10% of the recovered plasmid generated as a consequence of TLS. All TLS events observed in the *xpa*/*polζ* cells were associated with deletion at damaged sites (Figure 5D). Thus, as found previously [28] (Figure 5C), we conclude that Polζ is required for the successful bypass of T-T (6-4) UV photoproducts by TLS. Remarkably, the *xpa/polη/polζ* cells displayed a normal TLS efficiency, indicating that the failure of TLS in *polζ* cells is dependent on the presence of Polη.

We also classified the replication products obtained from the pQTo plasmid, where bypass can be effected only by TLS or deletion. As found in the previous study [28], the loss of Polζ was frequently associated with the deletion of two or more nucleotides covering the site of the T-T (6-4) UV photoproduct (Figure 5E and 5F). The loss of Polζ did not impair TLS in the absence of Polη, but severely compromised it in the presence of Polη. A possible explanation, discussed below, is that the Polη-dependent insertion of nucleotides opposite the T-T (6-4) UV photoproduct inhibits the completion of TLS in the absence of Polζ (presumably due to defective extension from inserted nucleotides), while other unidentified DNA polymerases can perform the complete TLS reaction in *polη/polζ* cells.

### Polκ plays only a minor role in the damage tolerance of *polη/polζ* cells

**Figure 6:**
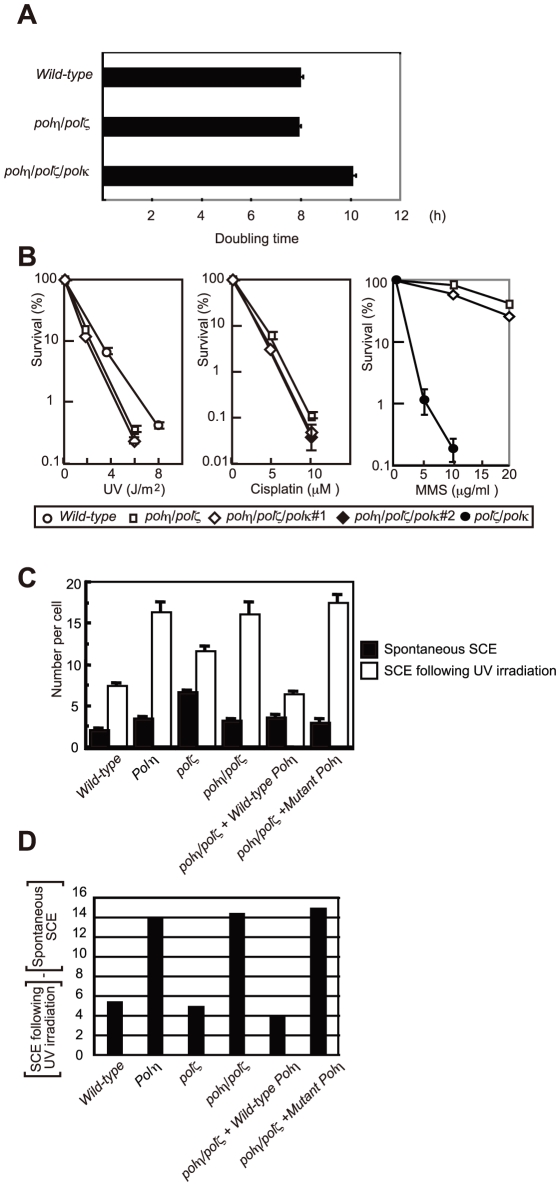
HR, but not Polκ, contributes to increased cellular tolerance to DNA damage in *polη/polζ* cells, compared with *polζ* cells. (A) Growth of *polη/polζ* and *polη/polζ/polκ* clones. Cells were cultured for 3 days. The doubling time of each mutant is shown. Error bars show the SD of the mean for three independent assays. (B) Sensitivity profiles of indicated mutant cells to UV and cisplatin. Error bars show the SD of the mean for three independent assays. (C) Induction of SCE in *polη*, *polζ*, and *polη/polζ* cells by UV. Cells were labeled with BrdU during over cell-cycles with or without UV irradiation (0.25 J/m^2^) before labeling. Spontaneous and induced SCEs in the macrochromosomes of 50 metaphase cells were counted. Histograms show the frequency of cells with the indicated numbers of SCEs per cell. The mean number of SCEs per cell ± SE is shown. Statistical significance was further calculated by the non-parametric Mann-Whitney U test. (D) The number of UV-induced SCE was calculated by subtracting spontaneous SCE from SCE following UV irradiation.

The milder phenotype of the *polη/polζ* cells, compared with *polζ* single mutants, led us to investigate the contribution of other TLS polymerases to TLS in *polη/polζ* cells. We previously showed that *polκ/polζ* cells show a higher sensitivity to mono-alkylating agents, compared with *polζ* cells, though *polκ* cells show normal sensitivity [36], and thereby suggested that Polκ can partially substitute for the loss of Polζ. We therefore sought to determine whether Polκ contributes to damage tolerance in *polη/polζ* cells. To this end, we deleted the *Polκ* gene in *polη/polζ* cells and analyzed the phenotype of the resulting triple knockout *polη/polκ/polζ* clones (Figure 6A). Deletion of the *Polκ* gene tended to reduce growth kinetics. However, the *polη/polκ/polζ* clones exhibited only limited increase in DNA-damage sensitivity, compared with *polη/polζ* cells (Figure 6B). Likewise, overexpression of chicken Polκ did not increase cisplatin tolerance in *polζ* or *polη/polζ* cells (data not shown). These observations imply that Polκ does not play an important role in TLS in *polη/polζ* cells.

### *polη/polζ* cells, but not *polζ* cells, displayed increased numbers of UV-induced sister chromatid exchange events

**Figure 7:**
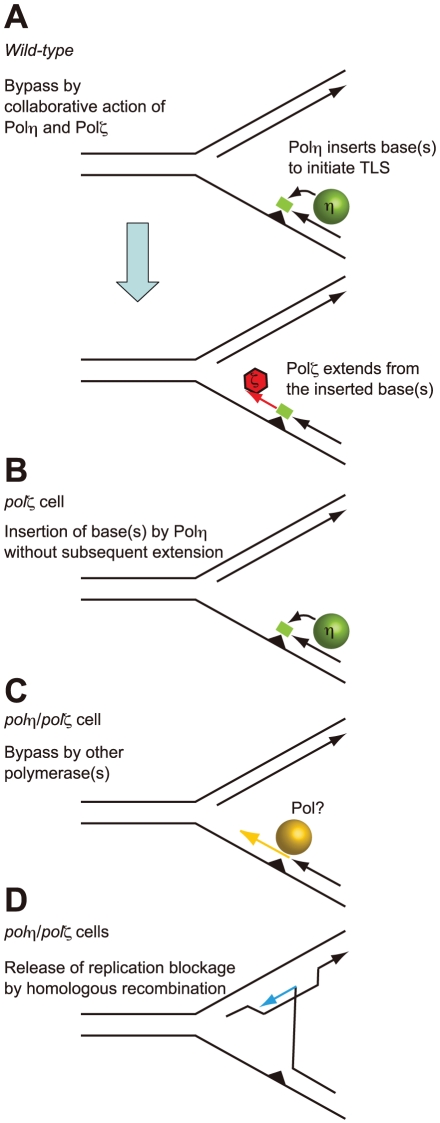
Model for the sequential action of Polη and Polζ in *wild-type*, *polζ*, and *polη/polζ* clones. (A) The two step-model for TLS. In *wild-type* cells, Polη is efficiently recruited to a replication blockage at a DNA lesion (green circle) on a template strand and inserts 1∼2 base(s) (green box) to bypass the lesion (filled triangle). Polζ further extends from these inserted base(s) (red arrow) before replicative DNA polymerases can take over from Polζ. (B) In *polζ* cells, after Polη inserts nucleotides opposite to DNA lesions, no other polymerases can effectively extend DNA synthesis for subsequent DNA synthesis by replicative polymerases. (C) In *polη/polζ* cells, other TLS polymerases carry out TLS. (D) The replication blockage may be released by a template switch to the intact sister chromatid using the HR machinery.

Replication arrest can be released by two major mechanisms: HR and TLS [37]. HR-dependent release is initiated by homologous pairing between the 3′ end of the arrested strand and the sister chromatid, followed by strand invasion and DNA synthesis to extend the invading 3′ strand (Figure 7D). To analyze the efficiency of HR-mediated release from replication blockage, we analyzed sister chromatid exchange (SCE) (Figure 6C and 6D) [38], [39]. The level of SCE is likely to be determined by two factors: the number of DNA lesions that cause a replication block and the efficiency of HR-dependent release from replication blockage. As previously reported [9], [39], the level of spontaneous SCE was slightly increased (1.5 to 2-fold) in all TLS mutants compared with *wild-type* cells, presumably because lesions are more frequently channeled to HR. The *polη* cells displayed a markedly greater increase in the level of UV-induced SCE (the number of spontaneous SCE subtracted from the number of SCE following UV irradiation shown in Figure 6D), which phenotype is attributable to defective TLS over UV-induced damage. In contrast, in the *polζ* cells, the UV-induced SCE level was similar to that of *wild-type* cells, suggesting that the defective TLS may not be adequately compensated by HR [9].

SCE was induced more efficiently in the *polη/polζ* cells than in the *polζ* cells, which is consistent with the increased UV tolerance of *polη/polζ* cells, compared with *polζ* cells. Reconstitution of the *polη/polζ* cells with *wild-type POLη*, but not with the catalytically inactive *POLη*, significantly reduced the number of UV-induced SCE events. Thus, the degree of UV tolerance correlated with the number of UV-induced SCE events at least in *polζ* and *polη/polζ* cells. This observation suggests that, in addition to TLS, HR-mediated release from replication blockages contributes to a significantly higher UV tolerance in *polη/polζ* cells than in *polζ* cells.

## Discussion

Polη is required for TLS across a much wider range of DNA lesions than indicated by the sensitivity of Polη-deficient cells. Analysis of XP-V cells indicates that Polη plays a major role in TLS past cyclobutane dimers and certain bulky adducts, but few other lesions. We demonstrate here that, in DT40 cells, Polη is also involved in the interaction of the replication machinery with different types of damage, such as those induced by chemical cross-linking agents (Figure 2), abasic sites (Figure 3 and Figure 4), and T-T(6-4) UV photoproduct (Figure 5). Thus, the deletion of *polη* in the *polζ* cells reversed their hypersensitivity to UV, MMS, cisplatin, and the ectopic expression of AID. Our finding that *polη/polζ* cells were significantly more tolerant to all tested DNA-damaging agents compared with *polζ* cells, reveals that neither of these polymerases is absolutely required for the tolerance of these types of damage during DNA replication. However, when Polη is present, Polζ is also required for efficient recovery from the effects of these damaging agents.

XP-V cells show a modest phenotype, including moderate sensitivity to UV and cisplatin [40], only in the presence of caffeine, and do not show significant sensitivity to MMS. Consequently, it was once believed that Polη does not play a role in TLS past DNA lesions induced by alkylating agents. However, *in vitro* biochemical studies have shown that purified Polη can bypass a variety of lesions including 7, 8-dihydro-8-oxoguanine (8-oxoG), *O^6^*-methylguanine, abasic sites, benzo pyrene adducts, and cisplatin intrastrand crosslinks [2], [41]–[43]. The present study helps demonstrate that Polη can indeed be involved in TLS past a wide variety of DNA lesions, even without caffeine treatment in DT40 cells. This idea is relevant to mammalian cells, since Polη focus formation is observed in mammalian cells following treatment with cisplatin and UV in the absence of caffeine [40].

### The collaborative action of Polη and Polζ in TLS

The improved damage tolerance of *polη/polζ* cells, compared with *polζ* cells, suggests following several possibilities. One possibility is that Polζ somehow inhibits Polη action and that Polη does not actually have a significant role when Polζ is present. However this possibility may be unlikely, since physical interaction between Polη and Rev1, and Polη dependent recruitment of Rev1 to the DNA damage site support the sequential actions of Polη followed by Polζ rather than inhibitory action of Polζ on Polη [44]–[47]. Thus, more likely possibility is that, Polη generates a replicative intermediate in an attempt to bypass the lesion, but cannot complete an effective bypass reaction without Polζ (Figure 7A). We suggest that the abortive intermediates generated by Polη in the absence of Polζ lead to a difficult-to-rescue replication collapse, thereby accounting for the hypersensitivity of the single *polζ* mutant (Figure 7B). The modest phenotype of XP-V cells indicates that Polζ may efficiently mediate TLS past a variety of DNA lesions, either in collaboration with other polymerases or possibly on its own.

This situation can be explored further in the light of current TLS models. In the canonical model for TLS replication, arrest by agents such as UV, MMS, and cisplatin, leads to PCNA becoming mono-ubiquitinated [40], [48]–[50]. This increases the affinity of PCNA for Polη and other Y-family TLS polymerases by virtue of their UBM and UBZ ubiquitin-binding motif [44], [48], [50]–[52] and likely contributes to the accumulation of Y-family polymerases at the sites of blocked replication forks. It has been suggested that these polymerases can compete with each other by mass action to attempt to carry out TLS. In the case of CPDs, if Polη wins this competition; bypass can occur without the need for a second polymerase. However, with other lesions such as a T-T (6-4) photoproduct, there is currently no evidence to show that any single polymerase can complete bypass. Polη may be able to start the bypass by incorporating opposite at least the 5′ base of the lesion, but it cannot extend from the resulting mismatch (Figure 7A). This would explain the abortive intermediate referred to above. To complete TLS, Polζ is required to extend from the inserted nucleotides (Figure 7A), an explanation that is consistent with the sequential action of Polη and Polζ demonstrated in *in vitro* studies [2]. A further implication of this model is that no other polymerase can effectively substitute for Polζ in this extension step (Figure 7B).

The successive action of Polη and Polζ might be mediated by the association of the two polymerases with Rev1 [45], [53]. The idea of a Rev1-mediated switch from Polη to Polζ is supported by the fact that Polη tightly interacts with Rev1 [1], [46], [47], [53], [54]. Moreover, DNA-damage-induced Rev1 focus formation appears to be dependent on Polη [46], [47]. Adding to these findings, the present work establishes a role for Polη in the bypass of a wider range of DNA damage than previously thought and demonstrates the *in vivo* importance of the two-step bypass of many lesions.

The effect on TLS of depleting both Polη and Polζ is distinctly different in DT40 cells than in human cells. Ziv et al. showed that depletion of Rev3 sensitized cells to UV more severely at two days after irradiation in Polη-deficient XPV fibroblasts than in Polη-proficient control cells [22], although its impact on Polη-proficient DT40 cells was considerably stronger than on Polη-deficient DT40 cells. There are several explanations for this apparent difference. First, we favor the idea that DT40 may be a more reliable cell line than others to evaluate TLS by measuring cellular survival due to following reasons. The cell cycle distribution is distinctly different between DT40 and other mammalian cell lines. In DT40 cells, ∼70% of the cells are in the S phase, and the G_1_/S checkpoint does not function at all [55]. In most of the mammalian cell lines, on the other hand, more than 50% of the cells are in the G_1_ phase, and G_1_/S checkpoint works at least partially. Therefore, environmental DNA damage interferes with DNA replication more significantly in DT40 cells than in mammalian cell lines. Accordingly, TLS contributes to the cellular survival of the colony formation assay to a considerably higher extent in DT40 cells than in mammalian cell lines. Ziv et al., on the other hand, evaluated TLS by measuring the number of living cells at 48 hours after UV irradiation [22]. Since a majority of UV irradiated cells may have stayed outside the S phase at 48 hours, it is unclear whether this survival reflects the efficiency of TLS. The same laboratory also analyzed TLS past a cisplatin G-G intrastrand crosslink located in a gapped episomal plasmid [21]. Depletion of Rev3 with or without codepletion of Polη in U2OS cells resulted in an 80% reduction in TLS past the lesion, irrespective of the presence or absence of Polη. The relevance of this finding to TLS during the replication of chromosomal DNA remains elusive. Yoon et al. investigated TLS in a double-stranded plasmid containing a single 6-4 photoproduct as well as replication origins derived from the SV40 virus [23]. Depletion of Rev3 or Rev7 in NER-deficient XP-A fibroblasts reduced the efficiency of TLS in the episomal plasmid by approximately 50%, with a similar reduction obtained in XP-V fibroblasts. This result using human fibroblasts is clearly different from the data we obtained using DT40 cells. Given the close sequence similarity between the two polymerases in human and chicken cells, we consider it unlikely that the mechanisms of lesion bypass are fundamentally different between the two organisms. The apparent difference between mammalian cells and DT40 may be caused by the incomplete si-RNA mediated inhibition in human cells versus the null mutation we have used in DT40. Another possible reason to explain this difference is the active HR system in DT40 cells, and the different usage order of TLS DNA polymerases because the DT40 B lymphocyte line undergoes Ig V hypermutation through TLS [32]. The usefulness of the three episomal plasmid systems to the analysis of TLS occurring during replication of chromosomal DNAs should be further investigated [15], [21], [22], [28]. Irrespective of the reason for this apparent difference, our data clearly indicate that, as discussed above, under some conditions Polη can hinder the efficient progress of the replication fork past lesions mediated by Polζ.

### Rescue of failed translesion synthesis by homologous recombination

It is remarkable that deletion of the three major TLS polymerase genes, *POLη*, *POLκ*, and *POLζ*, results in only a mild reduction in the growth kinetics of DT40 cells (Figure 6A). This is in marked contrast with the immediate cell death associated with the massive chromosomal breaks generated upon deletion of Rad51 [56]. These observations imply that during DNA replication, if replication blocks are encountered, HR can at least partially compensate for defective TLS. The significant functional redundancy between TLS and HR is supported by our previous report, which concludes that the deletion of both *RAD18* and *RAD54*, a gene involved in HR, as well as the deletion of both *REV3* and *RAD54*, are synthetically lethal to cells [9], [39]. We show here that depletion of Polη irrespective of the status of Polζ markedly increases the level of UV-induced SCE (Figure 6C), suggesting that DNA damage that cannot be resolved by TLS because of the absence of Polη may be resolved by HR, leading to increased SCE levels (Figure 7D). However, SCE is not induced to the same level in *polζ* cells (Figure 6C). Thus, nucleotide incorporation by Polη appears to represent a point of commitment in the TLS reaction beyond which rescue by HR is problematic. This is likely to be explained by the creation of an intermediate, possibly the mismatched primer terminus, which can be efficiently extended by Polζ, but which cannot readily initiate HR.

In summary, the significant increase in the cellular tolerance of *polη/polζ* cells to DNA-damaging agents, compared with *polζ* cells, can be partially attributable to more efficient HR in *polη/polζ* cells than in *polζ* cells. However, the normal level of TLS-dependent Ig V mutation and restoration of TLS during 6-4 photoproduct bypass in *polη/polζ* cells (Figure 4B) suggests that one or more other unidentified TLS polymerases can act as a substitute and carry out TLS when both Polη and Polζ are missing (Figure 7C).

## Materials and Methods

### Cell lines and cell culture

Generation of *polζ* (*rev3*)- and *polη*-deficient DT40 cells was described previously [9], [12]. To generate *polη/polζ* cells, we sequentially introduced two *rev3* gene-disruption constructs (*rev3-hygro* and *rev3-His*) [9] into *polη* (Puro^R^/Bls^R^) cells. A puromycin-resistant *XPA* disruption construct was used to disrupt the single *XPA* allele and recreate the *xpa/rev3* cell line. After removal of the Bls^R^ marker gene from the *polη/polζ* cells by the transient expression of CRE recombinase, a blasticidin-resistant *XPA* disruption construct was used to generate the *xpa*/*polζ/polη* cell line. A puromycin-resistant *POLκ* disruption construct was used to generate *polη/polζ/polκ* cells. To make a *POLη* expression plasmid, we inserted human *POLη* cDNA into the multi-cloning sites (MCS) of an expression vector, pCR3-*loxP*-MCS-IRES-GFP-*loxP* [57]. A mutant Polη that lacks polymerase activity (*Mutant POLη*) was generated by inserting D115A/E116A mutations into human *Polη* cDNA. The conditions for cell culture, selection, and DNA transfection were described previously[58]. The growth properties of cells were analyzed as described previously [58].

### AID overexpression by retrovirus infection

For the retrovirus infection, the pMSCV-IRES-GFP recombinant plasmid was constructed by ligating the 5.2 kb *Bam*HI-*Not*I fragment from pMSCVhyg (Clontech) with the 1.2 kb *Bam*HI-*Not*1 fragment from pIRES2-EGFP (Clontech). Mouse AID cDNA [33] was inserted between the *Bgl*II and *Eco*RI sites of pMSCV-IRES-GFP. The preparation and infection of the retrovirus were carried out as previously described [33]. Expression of the GFP was confirmed by flow cytometry. The efficiency of infection was more than 90% as assayed by GFP expression.

### Assay of TLS past a T-T (6–4) photoproduct on episomal plasmid

pQTs and pQTo plasmids containing a T-T (6-4) photoproduct were generated and transfected into DT40 cells as previously described [28].

### Chromosome aberration analysis

Karyotype analysis was performed as described previously [56]. Cells were treated with colcemid for 3 hours to enrich mitotic cells.

### Measurement of SCE level

Measurement of SCE level was performed as described previously [38]. For UV-induced SCE, cells were suspended in PBS and irradiated with 0.25 J/m^2^ UV followed by BrdU labeling.

### Ig V_λ_ mutation analysis

Genomic DNA was extracted at 14 days after subcloning. The rearranged V_λ_ segments were PCR amplified using 5′-CAGGAGCTCGCGGGGCCGTCACTGATTGCCG-3′ as the forward primer in the leader-V_λ_ intron and 5′-GCGCAAGCTTCCCCAGCCTGCCGCCAAGTCCAAG-3′ as the reverse primer in the 3′ of the JC_λ_ intron. To minimize PCR-introduced mutations, the high-fidelity polymerase, Phusion (Fynnzymes) was used for amplification (30 cycles at 94° C for 30 s; 60° C for 1 min; 72° C for 1 min). PCR products were cloned using a Zero Blunt TOPO PCR Cloning Kit (Invitrogen) and subjected to sequence analysis with the M13 forward (-20) or reverse primer. Sequence alignment using GENETYX-MAC (Software Development, Tokyo) allowed the identification of changes from the parental sequences in each clone.

As described previously [59], all sequence changes were assigned to one of three categories: point mutation, gene conversion, or ambiguous. This discrimination is based on the published sequences of V_λ_ pseudogenes that can act as donors for gene conversion. For each mutation, the database of V_λ_ pseudogenes was searched for a potential donor. If no pseudogene donor containing a string >9 bp could be found, the mutation was categorized as a non-templated point mutation. If such a string was identified and there were further mutations that could be explained by the same donor, then all these mutations were categorized as a single long-tract gene conversion event. If there were no further mutations, indicating that the isolated mutation could have arisen through a conversion mechanism or could have been non-templated, it was categorized as ambiguous.

## Supporting Information

Figure S1 Increased phospholylation of histone H2AX in polζ deficient cells. (A) Fluorescence image of fixed DT40 cells with indicated genotype. Cells were stained with antibody to phospho-Histone H2AX (red) and with DAPI (Blue). (B) Percentage of the cells with over 10 phospho histone H2AX signals was calculated.
(1.94 MB PDF)

Figure S2 Proliferation of wild-type DT 40 cells carrying human POLη transgene or control expression vector. (A) Expression of the human POLη transgene was examined by Northern blot analysis. The level of endogenously expressed human POLη in 293T cells was below the detection limit, while human POLη expression was detected in DT40 cells carrying the human POLη transgene, indicating that human POLη was overexpressed in DT40 cells. (B) Proliferation of cells was examined for 3 days. Ectopic expression of human POLη in wild-type DT40 cells has no impact on cell proliferation.
(1.13 MB PDF)
