# Supplementary figures and images for: Simultaneous Disruption of Two DNA Polymerases, Polη and Polζ, in Avian DT40 Cells Unmasks the Role of Polη in Cellular Response to Various DNA Lesions
Source: PLoS Genet. 2010 Oct 7;6(10):e1001151. doi: 10.1371/journal.pgen.1001151 (PMC2951353; doi:10.1371/journal.pgen.1001151)

Figure S1

**A**

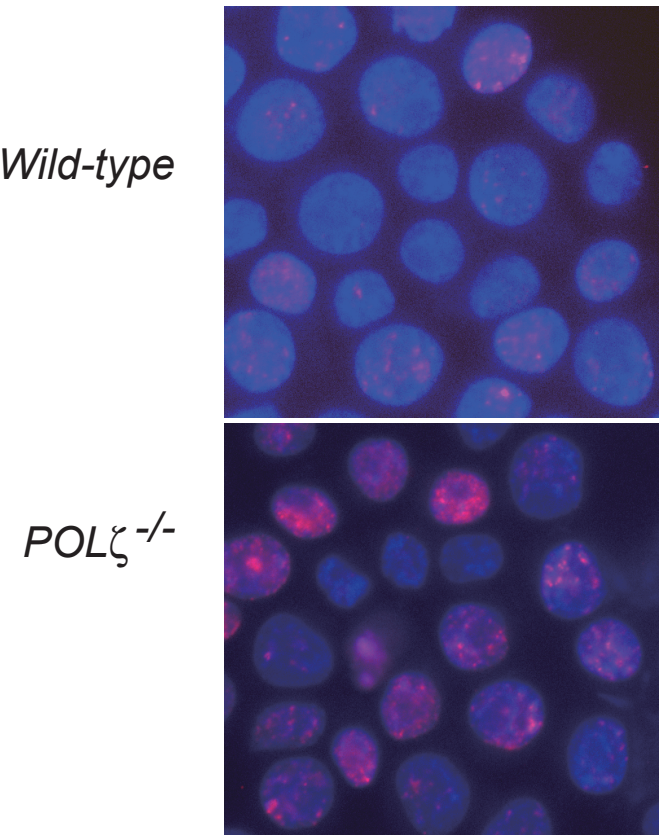

**B**

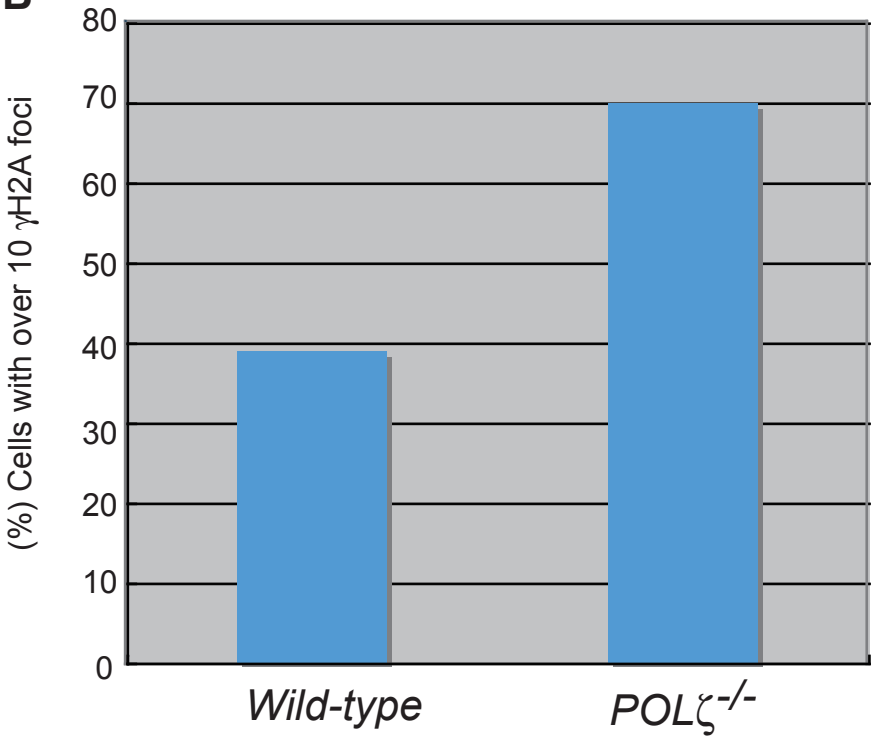

Supplement: Figure S1 — Increased phospholylation of histone H2AX in polζ deficient cells. (A) Fluorescence image of fixed DT40 cells with indicated genotype. Cells were stained with antibody to phospho-Histone H2AX (red) and with DAPI (Blue). (B) Percentage of the cells with over 10 phospho histone H2AX signals was calculated. (1.94 MB PDF) [file pgen.1001151.s001.pdf]

Figure S2

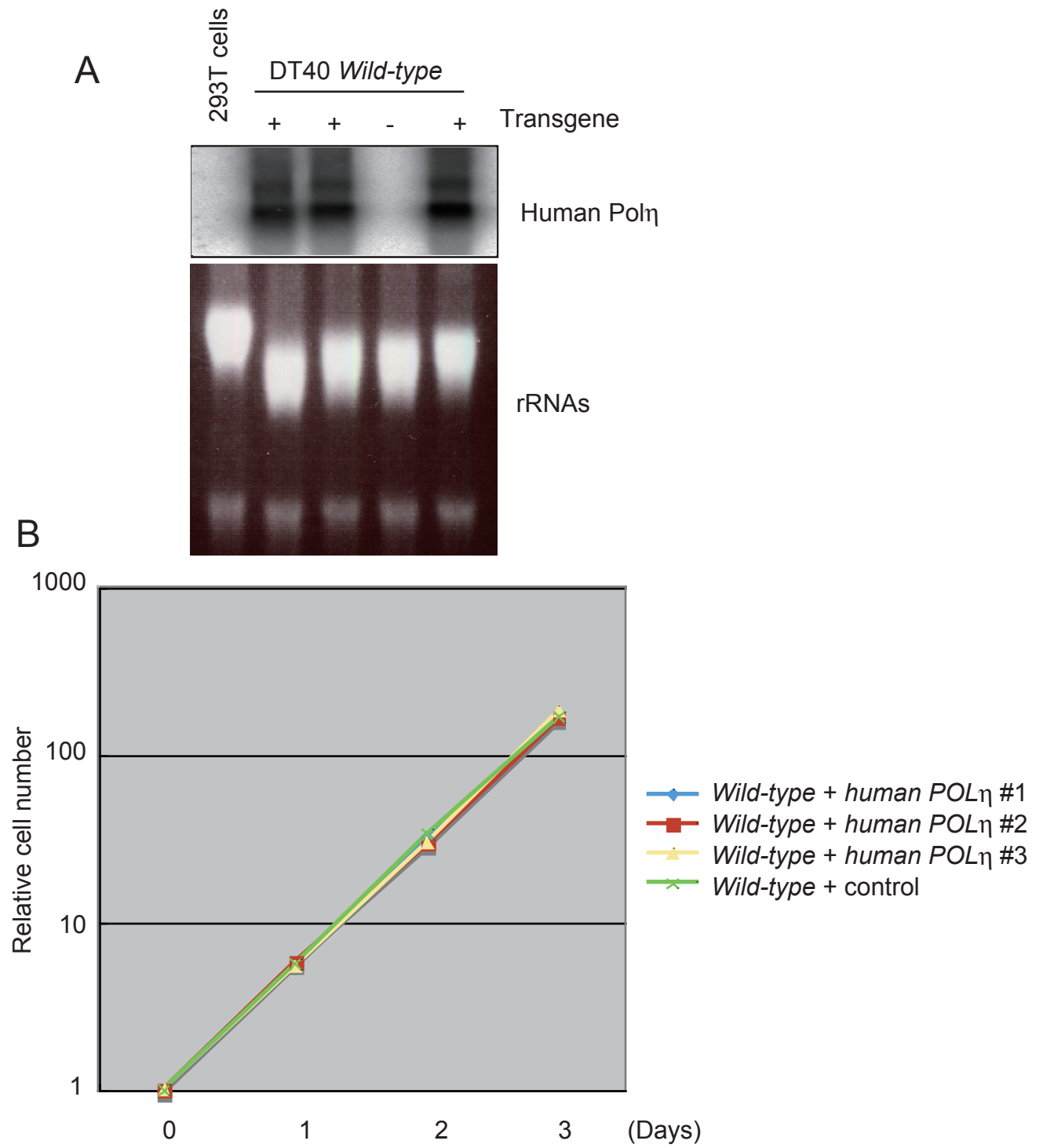

Supplement: Figure S2 — Proliferation of wild-type DT 40 cells carrying human POLη transgene or control expression vector. (A) Expression of the human POLη transgene was examined by Northern blot analysis. The level of endogenously expressed human POLη in 293T cells was below the detection limit, while human POLη expression was detected in DT40 cells carrying the human POLη transgene, indicating that human POLη was overexpressed in DT40 cells. (B) Proliferation of cells was examined for 3 days. Ectopic expression of human POLη in wild-type DT40 cells has no impact on cell proliferation. (1.13 MB PDF) [file pgen.1001151.s002.pdf]
